# Postoperative speech impairment and cranial nerve deficits in children undergoing posterior fossa tumor surgery with intraoperative MRI – a prospective multinational study

**DOI:** 10.1007/s00701-025-06669-3

**Published:** 2025-09-22

**Authors:** Aske Foldbjerg Laustsen, Jonathan Kjær Grønbæk, Radek Frič, Shivaram Avula, Conor Mallucci, Pelle Nilsson, Per Nyman, Péter Hauser, Katalin Mudra, Rosita Kiudeliene, Saulius Ročka, Magnus Aasved Hjort, Rick Brandsma, Eelco Hoving, Andrea Carai, Vladimír Beneš, Jana Táborská, Christian Dorfer, Sandra Jacobs, Miriam Pavon-Mengual, Jane Skjøth-Rasmussen, Kjeld Schmiegelow, Astrid Sehested, René Mathiasen, Marianne Juhler

**Affiliations:** 1 Department of Neurosurgery, Rigshospitalet, Copenhagen, Denmark; 2 Department of Pediatrics and Adolescent Medicine, Rigshospitalet, Copenhagen, Denmark; 3 Department of Neurosurgery, Oslo University Hospital – Rikshospitalet, Oslo, Norway; 4 Department of Radiology, Alder Hey Children’s NHS Foundation, Liverpool, UK; 5 Department of Neurosurgery, Alder Hey Children’s NHS Foundation, Liverpool, UK; 6 Department of Medical Sciences/Neurosurgery, Uppsala University Hospital, Uppsala University, Uppsala , Sweden; 7 Crown Princess Victoria Children’s Hospital and Department of Biomedical and Clinical Sciences, Linköping University, Linköping, Sweden; 8 2nd Department of Pediatrics, Semmelweis University, Budapest, Hungary; 9 Center of Pediatric Oncology and Hematology, Pediatric Department and Hospital of Kauno Klinikos, Lithuanian University of Health Sciences, Kaunas, Lithuania; 10 Clinic of Neurology and Neurosurgery, Faculty of Medicine, Vilnius University, Vilnius, Lithuania; 11 Department of Pediatric Hematology and Oncology, St Olavs Hospital, Trondheim, Norway; 12 Princess Maxima Center for Pediatric Oncology, Utrecht, Netherlands; 13 Neurosurgery Unit, Bambino Gesù Children’s Hospital, IRCCS, Rome, Italy; 14 Department of Neurosurgery, 2nd Medical Faculty, Motol University Hospital, Prague, Czechia; 15 Department of Neurosurgery, Medical University of Vienna, Vienna, Austria; 16 Pediatric Oncology, Department of Oncology, KU Leuven, Leuven, Belgium; 17 Neuro-Oncology Unit, Pediatric Cancer Center Barcelona, Hospital Sant Joan de Déu, Barcelona, Spain; 18 Department of Neurosurgery, Aarhus University Hospital, Aarhus, Denmark

**Keywords:** Posterior Fossa Tumor, Intraoperative Magnetic Resonance Imaging, Pediatric Neurosurgery, Cranial Nerve Deficits, Cerebellar mutism syndrome, Posterior Fossa syndrome

## Abstract

**Background:**

Postoperative speech impairment (POSI) and cranial nerve deficits (CND) are common complications of pediatric posterior fossa (PF) tumor surgery. Intraoperative MRI (ioMRI) has proven a useful tool in achieving gross total resection. The risk of POSI and CND with ioMRI remains unclear, making it the primary scope of this study. Additionally, we assessed whether POSI was associated with CND.

**Methods:**

We prospectively included pediatric patients undergoing PF tumor surgery in 36 centers across 15 European countries. Neurological status and speech were assessed preoperatively and 1–4 weeks postoperatively. Surgical details, including tumor location and use of ioMRI, were recorded within 72 h of surgery. Postoperative CND were categorized as 0, 1, 2, or ≥ 3 nerves affected; POSI as habitual, reduced speech, or mutism. Proportional odds models estimated odds ratios (OR) for 1) POSI with stepwise adjustment for tumor location and age, and 2) CND with adjustment for preoperative CND and tumor location. Subgroup analyses assessed systematic differences, missing data, center-level effects, and histology adjustment.

**Results:**

Of 790 primary PF tumor surgeries, 141 (18%) involved ioMRI. POSI occurred in 183/790 (23%) and postoperative CND in 213/790 (27%). POSI-risk with ioMRI showed non-significant unadjusted OR (95% CI) 0.83 (0.53;1.30); adjusted OR 0.76 (0.43;1.35). Fewer CNDs were observed with ioMRI (unadjusted OR 0.63 (0.40;1.00), adjusted OR 0.58 (0.33;0.94), p = 0.03). POSI-risk was associated with more CNDs (adjusted OR for 1 CND: 2.06 (1.15;3.68); 2 CND: 2.13 (1.02;4.42); ≥ 3 CND: 4.15 (1.98;8.70), p < 0.05).

**Conclusions:**

ioMRI was not associated with increased risk of postoperative complications in this multicenter cohort. The reduction in CND among ioMRI cases may reflect derived effects on surgical decision-making, expertise, case-load and case-mix. Results should be interpreted with caution due to limited intraoperative data. The association between POSI-risk and cumulative CND may indicate extensive brainstem involvement. Our findings highlight the need to further explore how ioMRI-guided strategies affect functional outcomes in pediatric PF tumour surgery.

**Clinical Trials ID:**

NCT02300766 (October 2014)

**Supplementary Information:**

The online version contains supplementary material available at 10.1007/s00701-025-06669-3.

## Introduction

Gross total resection (GTR) of brain tumors is faced with a delicate balance between the benefit of improved progression-free and overall survival rates [20] versus the risk of aggressive resection resulting in complications by damage to central nervous system functions. For pediatric posterior fossa (PF) tumors, these complications involve lesions to the cerebellum or brainstem for surgery on midline tumors or cranial nerves for laterally located tumors. Cerebellar mutism syndrome (CMS) is a frequent and severe complication following surgery for PF tumors. The primary component of CMS is postoperative speech impairment (POSI), defined as symptoms of either severely reduced speech or absence of speech (mutism) [13, 21, 25]. Tumors in juxtaposition to or with an invasion zone in the brainstem carry both a risk of CMS [24], and a risk of damage to cranial nerve nuclei and brainstem tracts. Motor deficits following PF surgery may therefore result from lesions in the cerebellum, cerebellar peduncles, and brainstem tracts, whereas cranial nerve deficits (CNDs) result from damage to cranial nerve nuclei or the cranial nerves exiting from the brainstem. While damage to structures such as the cerebellar peduncles seems to be associated with POSI by the proximal disruption of cerebellar-cerebral outflow tracts [4, 23, 24], the association between POSI and CND as an indicator of brainstem damage is less clear.

Since early 2000, intraoperative magnetic resonance imaging (ioMRI) has been applied as a tool in pediatric neurooncological surgery [18]. During the last decade, an increasing number of centers have introduced ioMRI in neurosurgical procedures. ioMRI offers several advantages, including updated tumor localization, quantification of the extent of resection, and detection of early complications [5, 16]. Studies on ioMRI in pediatric neurooncological surgery are characterized by having a predominant focus on the extent of tumor resection [10, 15, 18, 27, 31]. The impact of ioMRI on resection and postoperative complications was previously evaluated on 584 pediatric brain tumor patients; this study revealed a substantial improvement in the likelihood of achieving GTR in pediatric neurooncological surgery, while postoperative complications remained within acceptable levels [29]. Despite the acknowledged potential of ioMRI in maximizing the extent of resection in pediatric PF tumor surgery [28], few studies have investigated how this is balanced against the risk of neurological complications. This is particularly relevant given the surgical access in PF tumor surgery, where the ventral part of the tumor and its location relative to the brainstem are largely obscured to the surgeon.

Although one previous study did investigate ioMRI findings that could predict the development of CMS [3], no prior prospective large-scale multinational study has undertaken a systematic assessment of risks of POSI and/or brainstem damage reflected by CND in ioMRI-assisted surgical interventions for pediatric PF tumors. We conducted an observational study based on the large cohort of children operated for PF tumors included in the European CMS Study [30], comparing the risk of POSI and CND in children operated with versus without the assistance of ioMRI.

## Methods and materials

### Study design

The”European study of the cerebellar mutism syndrome in children with brain tumors of the posterior fossa” (The European CMS study) is an observational, prospective, multicenter cohort study with the aim of studying CMS. The Research Ethics Committee of the Capital Region (H-6–2014-002) approved the study in Denmark. The study design has previously been published [30]. The risk of POSI or CND when utilizing ioMRI was not the primary outcome of the protocol, but the extensive data collection from this unique cohort allowed for investigating additional aspects of surgical complications.

### Participants

We prospectively included children below 18 years of age who underwent open surgery for a PF tumor between 2014 and 2025 in one of the 15 participating countries. Prior to enrolment, we obtained informed consent. The study design allowed for postoperative inclusion in case of emergency surgery, in which the patients were enrolled within seven days after surgery.

### Data collection

Patients were assessed preoperatively with a standardized neurological examination and speech assessment by a pediatrician or neurosurgeon, and their relevant medical history was documented. Within 72 h of surgery, the operating neurosurgeon recorded the tumor location and use of ioMRI in a previously published surgical form [13]. No MRI data were available regarding preoperative knowledge of tumor location; therefore, we used the tumor location registered by the neurosurgeon as a proxy variable when evaluating the potential confounding by tumor location. The decision to employ ioMRI was made in accordance with local standards in centers with access to ioMRI; no data were available regarding the reason for applying or not applying ioMRI. Postoperative standardized neurological examination and speech assessment were performed within two weeks of surgery by a pediatrician or a neurosurgeon. All data were entered into a secure online database.

Speech was recorded as ‘yes’ or ‘no’ to mutism during the postoperative period, and if ‘no’, a panel opened with ‘yes’ or ‘no’ to reduced speech during the postoperative period. If the observer registered ‘no’ to both mutism and reduced speech, the speech was considered habitual during the postoperative period. Cranial nerve impairment was registered by ‘yes’ or ‘no’. If any CND was present, an additional panel opened with tick boxes, 'right' and 'left', for each of the specific CN. Dysfunction was ticked off for right side and/or left side for each CN. For CNs where neither 'right' nor 'left' was ticked, the nerves were assumed to function normally. The severity of the specific CND was not reported.

### Statistical analysis

Statistical analyses were performed in R-studio (v. 2023.06.1). Results were presented as (proportional) odds ratio (OR) estimates with nominal 95% confidence intervals (95% CI), that is, we did not perform any adjustment for multiple tests. No imputation was performed, that is, all analyses were performed as 'complete case' analyses. The consequence of excluding data due to missing values for a potential confounder was investigated by applying the model without the adjustment for the confounder to the dataset, including only patients with non-missing values of the potential confounder and evaluating whether the results differed from the estimated results presented in the tables for the larger dataset. No differences of relevance for the conclusions were found.

The proportional odds assumption (POA) implies that the relationship between predictor variables and the cumulative odds of being in a higher severity category is consistent across all outcome thresholds. The POA was investigated graphically for the association between the predictor and the outcome by comparing the estimated OR from the proportional odds model to the estimated ORs from the corresponding ordinary logistic regression models. The graphical evaluation showed internal consistency between the estimates of the proportional odds model and the ordinary logistic regression models for both the unadjusted and adjusted models.

Furthermore, we did a robustness analysis looking only at patients operated within 14 days from diagnosis, where we further adjusted for the time between diagnosis and operation as a categorical variable, categorized as 0, 1, 2, 3 or more days. This analysis was performed to get an indication of whether the association with ioMRI was partially caused by an association with the urgency of the operation. This proved not to be the case.

#### POSI

Postoperative speech status was categorized by a previously published hierarchical structure: mutism, reduced speech and habitual speech [13]. This hierarchy assumes that mutism and reduced speech reflect a continuum of severity within the same syndrome and adheres to the consensus definition of speech impairment in CMS [14].

#### Cranial nerve deficit classification

We evaluated the total number of postoperative damages to CN III through CN XII categorized into four categories (0, 1, 2 and 3 or more – Supplementary Table 1–4) We investigated the association of the number of postoperative CND with ioMRI univariately, adjusted for the number of preoperative CND as a linear variable, as well as further adjusted for tumor location (Supplementary Table 5).

To examine the association between ioMRI and potential postoperative change in cranial nerve involvement, we performed supplementary analyses of the change in the total number of CND from pre- to post-operation, subtracting the total number of preoperative CND from the total number of postoperative CND and categorizing them into − 2 or less, − 1, 0, 2, 3 and 4 or more). The POA was less appropriate for the univariate analysis using this categorical change variable as the outcome (Supplementary Table 6).

We considered adjusting for intraoperative neuromonitoring of cranial nerves in combination with ioMRI, but the subgroup comprised only 15 patients. Due to limited statistical power, it was not included in the multivariate analyses.

#### Tumor location, tumor histology and age

As the parameters have previously been documented as non-modifiable factors for POSI-risk, the statistical analyses were adjusted for these. Tumor location was grouped into four mutually exclusive categories based on tumor involvement of the anatomical structures: 1) Brainstem, 2) 4th ventricle without brainstem, 3) Vermis without brainstem or 4th ventricle and 4) cerebellar hemisphere only. Tumor histology was registered as pilocytic astrocytoma, medulloblastoma, ependymoma, atypical teratoid/rhabdoid tumor and other. Analyses adjusting for tumor histology made no substantial difference to the results and were included in *supplementary material*. Age-adjusted analyses were performed using age as a continuous variable and as a linear spline with knots (age groups: 0–3, 3–7, and > 7 years). The continuous variable provided a better fit to the data and was used in the final models.

#### Geographical impact

Because 7 out of 15 countries had applied ioMRI during the inclusion period, we performed analyses adjusting for country effect and analyses on a subset only including countries with access to ioMRI, which did not change the overall conclusions (Supplementary Table 7–10).

## Results

**Fig. 1:**
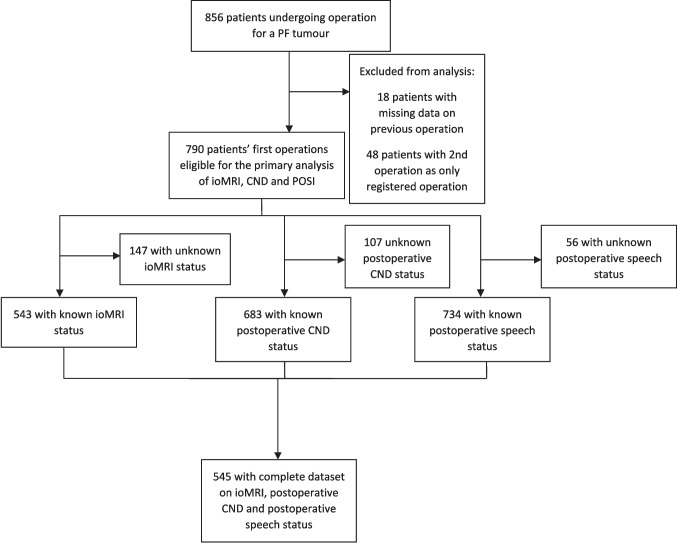
Flowchart for patient inclusion

We enrolled a total of 856 patients undergoing PF tumor operation. We excluded 18 patients due to missing data on previous tumor operation as well as 48 patients due to their second operation being registered as their only operation. The exclusion left 790 patients with primary tumor operation for analysis (Fig. 1). Table 1 shows the demographics of the 790 patients (detailed demographics tables including cross table between POSI and postoperative grouped CND can be found in Supplementary Table 11–12). The median age at operation was 7.0 years, with an interquartile range of 3.9 to 11.0 years. Of the 790 patients, 448 (57%) were male. The status of ioMRI utilization was known for 643/790 patients (81%), of which 141/643 (22%) were operated with the use of ioMRI. A total of 10 out of 36 centers in 7 out of 15 countries applied ioMRI during the inclusion period.

The tumor location based on the mutually exclusive categories involved fourth ventricle in 266/790 (34%), cerebellar hemisphere in 182/790 (23%), brainstem in 156/790 (21%), and vermis in 136/790 (17%), while not reported in 50 (6%).

POSI was observed in 183/790 (23%) of the study population, with 98/790 (12%) having reduced speech and 85/790 (11%) having mutism. Postoperative speech status was not reported for 56/790 patients (7%). Preoperative CND was registered in 132 patients (17%) and postoperative CND in 213 patients (27%), respectively. CN III, IV, VI, and VII were the most frequently affected (Supplementary Fig. 1–4).

Missing data were primarily due to insufficient data registration and, to a lesser extent, due to follow-up registration in progress, as the study continues to recruit patients and collect data.

**Table 1:** Cohort characteristics

Characteristic	All patients (n = 790)	Known status of Intraoperative MRI (n = 643)	Intraoperative MRI applied (n = 141)
Median age [IQR]	7.0 [3.9;11.0]	7.0 [3.9;10.8]	7.2 [4.2;10.6]
	N (*%*)	N (*%*)	N (*%*)
Sex			
Male	448 (*57*)	366 *(82)*	79 *(22)*
Female	342 (*43*)	277 *(81)*	62 *(22)*
Tumor location^a^			
Brainstem	156 (*20*)	143 *(87)*	39 *(27)*
4th ventricle	266 (*34*)	226 *(85)*	49 *(22)*
Vermis	136 (*17*)	108 *(79)*	17 *(16)*
Cerebellar hemisphere	182 (*23*)	146 *(80)*	30 *(21)*
Unknown	50 (*6*)	20 *(40)*	6 *(30)*
Postoperative speech impairment			
Habitual	551 (70)	444 *(81)*	103 *(23)*
Reduced speech	98 (*12*)	87 *(89)*	12 *(14)*
Mutism	85 (*11*)	71 *(84)*	18 *(25)*
Unknown	56 (*7*)	41 *(73)*	8 *(20)*
Preoperative accumulated CND^b^			
0	580 (*73*)	489 *(84)*	112 *(23)*
1	80 (*10*)	66 *(83)*	12 *(18)*
2	35 (5)	26 *(74)*	4 *(15)*
3	6 (*0.6*)	6 *(100)*	0
4	5 (*0.6*)	3 *(60)*	0
5	2 (*0.3*)	1 *(50)*	0
6	2 (*0.3*)	0	0
9	1 (*0.1*)	0	0
12	1 (*0.1*)	1 *(100)*	0
Unknown	78 (*10*)	51 *(65)*	13 *(26)*
Postoperative accumulated CND^c^			
0	470 (60)	387 *(82)*	91 *(24)*
1	89 (11)	83 *(93)*	12 *(14)*
2	65 (*8*)	51 *(78)*	9 *(18)*
3	22 (*3*)	16 *(73)*	4 *(24I*
4	9 (1)	8 *(89)*	1 *(13)*
5	6 (*0.8*)	4 *(67)*	0
6	8 (1)	5 *(63)*	1 *(20)*
7	3 (*0.4*)	1 *(33)*	0
8	4 (*0.5*)	4 *(100)*	1 *(25)*
10	4 (*0.5*)	3 *(75)*	0
12	1 (*0.1*)	1 *(100)*	0
13	1 (*0.1*)	0	0
18	1 (*0.1*)	0	0
Unknown	107 (*13.5*)	80 *(75)*	22 *(28)*

Column 1: total cohort, N *(%)*,

Column 2: excluding unknown ioMRI status, N *(%, of total cohort, row-wise)*,

Column 3: surgery with ioMRI, N *(%, of known ioMRI status, row-wise)*,

^a^Brainstem, 4th ventricle without brainstem involvement, Vermis without brainstem or 4th ventricle involvement and cerebellar hemisphere without brainstem, 4th ventricle or vermis involvement,

^b^Accumulated number of CN’s damaged preoperatively,

^c^Accumulated number of CN’s damaged postoperatively *CND* Cranial nerve deficit, *IQR* Interquartile range, *MRI* Magnetic resonance imaging, *N* Number

**Fig. 2:**
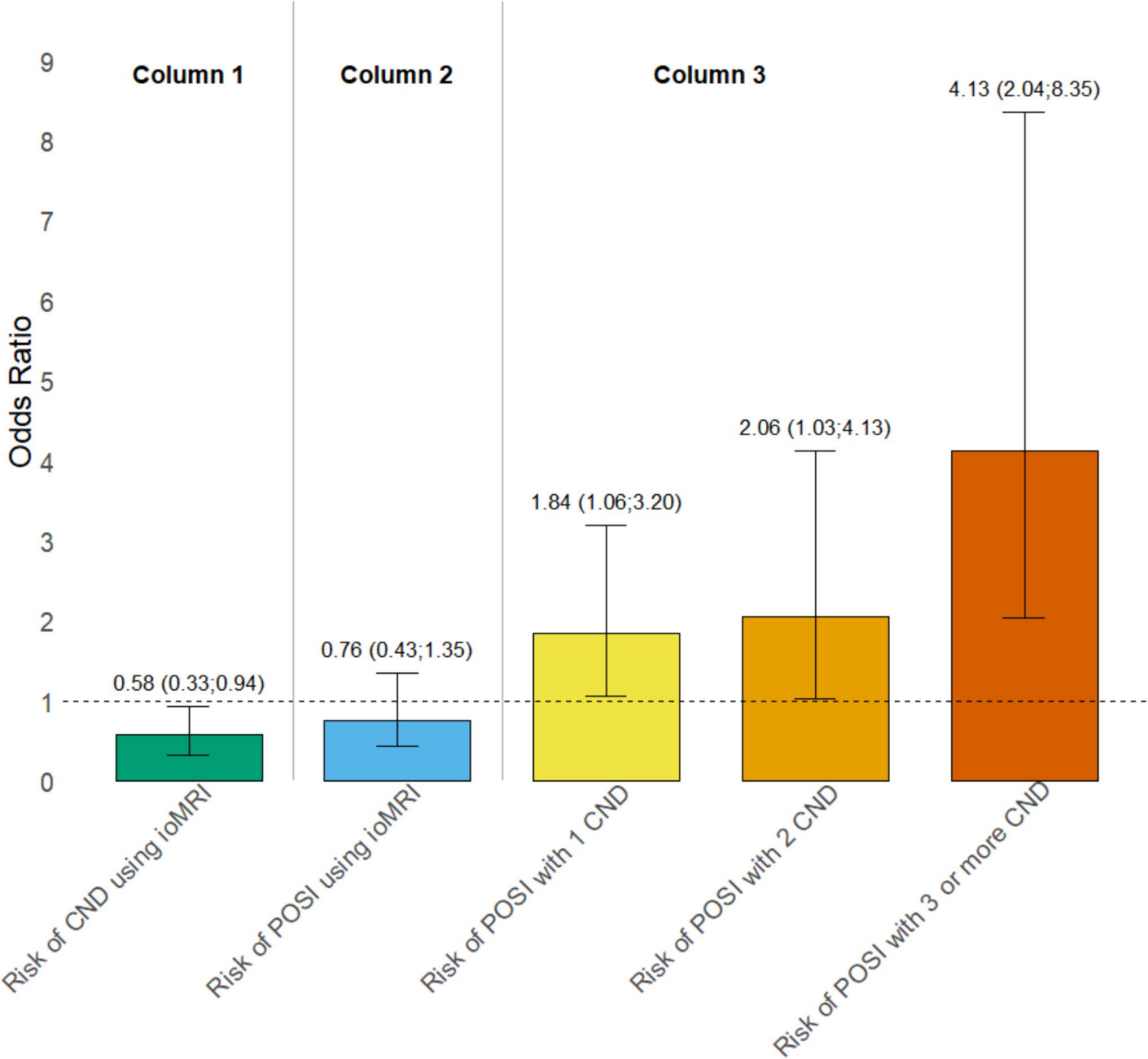
Odds Ratio results from the multivariate analyses; Odds ratio (95% confidence interval); column 1: CND and ioMRI, column 2: POSI and ioMRI, column 3: POSI and CND

Table 2 presents the univariate and multivariate analyses of POSI with ioMRI and postoperative CND as predictors. The analyses with ioMRI as predictor showed a tendency toward a decrease in the risk of POSI when utilizing ioMRI corresponding to an OR of 0.83 (95% CI: 0.53;1.30) in the univariate analysis and an OR of 0.76 (95% CI: 0.43;1.35) in the multivariate analysis, although non-significant (Fig. 2, column 2). The analyses with postoperative CND as predictor indicated that the presence of postoperative CND was associated with an increased risk in POSI corresponding to an OR of 2.90 (95% CI: 1.75;4.78) for 1 CND, 2.36 (95% CI: 1.31;4.26) for 2 CND and 5.39 (95% CI: 3.09;9.42) for 3 or more CND in the univariate analysis, and an OR of 1.84 (95% CI: 1.06;3.20) for 1 CND, 2.06 (95% CI: 1.03;4.13) for 2 CND, and 4.13 (95% CI: 2.04;8.35) for 3 or more CND in the multivariate analysis (Fig. 2, column 3).

**Table 2:** Risk of postoperative speech impairment

Univariate analysis		Multivariate analysis		
		Model 1^c^ (n = 545)	Model 2^d^ (n = 525)	Model 3^e^ (n = 523)
Intraoperative MRI^a^	0.83 (0.53;1.30) (n = 602)	0.85 (0.50;1.45)	0.73 (0.42;1.29)	0.76 (0.43;1.35) p = 0.36
Postoperative CND^b^	(n = 661)			
1	2.90 (1.75;4.78)	2.78 (1.65;4.70)	1.89 (1.09;3.27)	1.84 (1.06;3.20) p = 0.03
2	2.36 (1.31;4.26)	2.38 (1.23;4.55)	2.41 (1.09;5.33)	2.06 (1.03;4.13) p = 0.04
3 or more	5.39 (3.09;9.42)	4.72 (2.48;9.00)	3.53 (1.77;7.03)	4.13 (2.04;8.35) p < 0.0001

^a^Odds ratio (95% confidence interval) for POSI with “No ioMRI” as reference,

^b^Odds ratio (95% confidence interval) for POSI with “0 postoperative CND” as reference,

^c^Mutually adjusted with Intraoperative MRI & postoperative CND,

^d^As Model 1 + adjusted for tumour location, ^e^As Model 2 + adjusted for age *CND* Cranial nerve deficit, *MRI* Magnetic resonance imaging, *N* Number

Table 3 presents the analyses of the number of postoperative CND, categorized into 0, 1, 2, and 3 or more CNs affected, and ioMRI. The univariate analysis showed that the use of ioMRI was associated with a decrease in the number of postoperative CND with an OR of 0.63 (95% CI: 0.40;1.00). Adjustment for the number of preoperative CND and further adjustment for tumor location made only little difference (OR 0.58, 95% CI: 0.33;0.94) (Fig. 2, column 1).

**Table 3:** Risk of postoperative cranial nerve deficit

Univariate analysis		Multivariate analysis	
	(n = 563)	Model 1^b^ (n = 530)	Model 2^c^ (n = 513)
Intraoperative MRI^a^	0.63 (0.40;1.00) p < 0.05	0.67 (0.41;1.09)	0.58 (0.33;0.94) p = 0.03

^a^Odds ratio (95% confidence interval) for postoperative CND with “No ioMRI” as reference,

^b^Adjusted for preoperative CND,

^c^Adjusted for preoperative CND & tumour location *CND* Cranial nerve deficit, *MRI* Magnetic resonance imaging, *N* Number

## Discussion

Our results showed no association between use of ioMRI and significant changes in risk of POSI. Our observation of fewer CNDs aligns with previous studies reporting reduced neurological deficits associated with ioMRI use [29]. However, these findings do not allow a conclusion of direct or indirect causality. They are likely to represent derived effects by other surgical factors, such as pre- and intraoperative strategies or neurosurgical techniques aimed at functional preservation. It is also possible that ioMRI availability coincides with surgical expertise, higher patient volume and institutional protocols. Considering the balance between extent of resection and functional outcome, the presence of ioMRI may facilitate increased safety in functional preservation. However, in current surgical practice, ioMRI is typically used only once during the procedure and does not provide continuous, real-time guidance. Intraoperative neuromonitoring (ioNM) of the brainstem serves as complementary surgical tool by offering continuous real-time feedback on CN function, though its ability for detecting CN nuclei damage comes with restrictions. First, PF tumors impinging upon the dorsal brainstem often distort anatomical landmarks such as the facial colliculus and striae medullaris, introducing technical challenges with accurate identification of CN motor nuclei. Second, preserved intraoperative evoked potentials do not preclude postoperative CND, as complications such as delayed ischemia or vasogenic oedema may result in impaired CN nuclei function post-surgery [8]. These limitations highlight the constraints of ioNM in preventing CND when surgery involves close proximity to CN nuclei.

Our dataset did not include neither the necessary detail nor statistical power to account for these factors; however, overall surgeon experience has previously been associated with degree of morbidity in pediatric neuro-oncological surgery, including posterior fossa syndrome [2, 17]. Although not evident in our multicenter cohort, other institutions using ioMRI may also apply advanced neuronavigation, tractography, and ioNM, supported by institutional experience, to reduce the risk of postoperative functional deficits. These combined potential drivers of improved functional outcome should be addressed in future studies.

The observed association between increased POSI risk with increasing number of postoperative CND likely reflects the extent of brainstem involvement. More cranial nerve involvement would imply more brainstem involvement, explaining – to some extent – this association. These observations thus indirectly support an association between POSI and lesions to the brainstem tracts.

While CND is diagnosed with objective criteria in a structured neurological examination [7], POSI can be challenging to assess due to its multifaceted origins in brain function, affecting language production. These origins include 1) intention to speak, 2) word selection, 3) sentence formulation, 4) neural signaling from the cortex to the muscles within the oral cavity, pharynx, larynx, and respiratory system required for articulating the verbal output [19]. The POSI group in our study may be diverse concerning the underlying brain damage, resulting in speech impairment, manifesting as either reduced speech or mutism. This diversity can be attributed to damage on different brain levels. Simplified, speech impairment can originate from either “lower brain level” damage resulting in CND and, subsequently, coherent muscle movement deficiency [9], or”higher brain level” damage involving cognitive processes from volitional speech to sentence formulation. While lower-level function primarily depends on CN integrity, higher-level processes are thought to rely more on cerebello-cerebral connectivity involving language-related cortical areas [1, 22]. We consider POSI, in the context of CMS, to reflect a "higher brain level" impairment; however, since specific CND can also lead to speech deficits, misclassification is likely within our multi-observer setup, where each observer assessed a relatively small number of patients during the study period. Thus, the association between increasing CNDs and POSI risk could be impacted by a subset of patients with isolated "lower brain level" speech impairments, which would arguably not reflect the type of speech impairment traditionally considered the core symptom of CMS. Some CNDs (particularly of the CN V, VII, IX, X and XII) [26] are likely to be associated with speech impairment consistent with the aforementioned “lower brain level”. Being able to differentiate between CND relevant for speech production and CND irrelevant for speech production would bring further understanding to the POSI subgroups and the underlying pathophysiology of CMS. A potential way to differentiate POSI related to higher cognitive dysfunction from speech impairment secondary to lower CND is to consider differences in recovery time. Speech impairment in CMS often resolves within weeks [13], whereas impairment in verbal output caused by CND may last for months, possibly being a permanent state [6]. This would require addressing recovery trajectories of POSI and CND, which was not the scope of this study. In this context, it is possible that our finding of a higher incidence of POSI in association with CND partly reflect overestimation or suboptimal classification of POSI within our cohort. In general, additional factors potentially influencing POSI risk must be considered alongside the effect of tumor location and type [11, 12]. In a previous study, the apparent impact of modifiable factors – such as surgical technique – weakened considerably after adjusting for these tumor characteristics [13].

### Limitations

Our study is limited by not including the surgeon’s intent of utilizing ioMRI during surgery or documentation of the extent of resection quantified on postoperative MRI, both of which can be expected to influence risks and safety associated with an aggressive approach to obtain GTR.

It is also possible that preoperative factors, which could affect surgical outcome, might have influenced the decision or the access to use ioMRI, e.g., preference to apply ioMRI in extensive tumors with anatomically intricate locations and conversely, reduced practical applicability of ioMRI in emergency cases.

Missing data may have introduced a selection bias in context with speech outcomes. Loss to follow-up may have been more frequent in children with non-impaired speech due to the study’s focus on identifying POSI. Additionally, challenges with assessing speech in the youngest age group could both lead to an over- and underestimation of POSI in this subgroup.

Given the rarity of this condition, a multicentric design was necessary to ensure feasibility in patient numbers, which inherently introduces variability in surgical experience and treatment approaches between recruiting centers. Standardized reporting formats on outcomes and covariates were applied to reduce heterogeneity, and variation was further adjusted for on a country level, partly addressing this variability. Notably, the observed POSI incidence aligns with previously published data, and the multicenter setup lends some degree of overall external validity to the study results.

Our study lacked statistical power to analyze the association between individual or bilateral CND and POSI. We observed deficits in CNs III, IV, VI, and VII as the most common. This pattern can be partly explained by these nerve functions being the most apparent to assess by clinical examination in children — through eye movements and facial motor function — which may lead to higher detection rates. Additionally, motor cranial nerve nuclei develop medially in the dorsal brainstem, whereas sensory nuclei are generally located more laterally. The reticular formation, a longitudinal network located primarily in the dorsal brainstem, also plays a key role in coordinating both autonomic and motor functions, including cranial nerve activity. Since 4th ventricle tumor location was the most common in our study, and these often adhere to the posterior wall of the brainstem, surgical resection in this region may likely affect cranial nerve motor nuclei and/or parts of the reticular formation, further contributing to the pattern observed in our cohort.

## Conclusion

ioMRI was not associated with an increased risk of postoperative complications in this multicenter cohort. The observed reduction in CND among ioMRI cases, may reflect derived effects on surgical decision-making, expertise, case-load and case-mix. These findings should be interpreted with caution due to the lack of detailed intraoperative data. The observed association between the risk of POSI and cumulative postoperative CND may indicate more extensive involvement of brainstem structures. Our findings highlight the need for future studies to explore how ioMRI-guided surgical strategies influence functional outcomes in pediatric PF tumor surgery.

## Supplementary Information

Below is the link to the electronic supplementary material.

Supplementary material 1 (PDF 482 KB)

## Data Availability

The data supporting the findings of this study are derived from the ongoing European CMS study, which is currently in the recruitment phase. The dataset is not publicly available due to the confidential nature of patient data and the need to protect study participant privacy. Anonymized data can be made available upon reasonable request to the corresponding author, pending completion of recruitment and data consolidation, and in compliance with ethical and institutional guidelines.

## Abbreviations

CI: Confidence interval
CMS: Cerebellar mutism syndrome
CN: Cranial nerve
CND: Cranial nerve deficit
GTR: Gross total resection
ioMRI: Intraoperative magnetic resonance imaging
ioNM: Intraoperative neuromonitoring
OR: Odds ratio
PF: Posterior fossa
POA: Proportional odds assumption
POSI: Postoperative speech impairment
