# Supplementary material for: Postoperative speech impairment and cranial nerve deficits in children undergoing posterior fossa tumor surgery with intraoperative MRI – a prospective multinational study
Source: Acta Neurochir (Wien). 2025 Sep 22;167(1):252. doi: 10.1007/s00701-025-06669-3 (PMC12454580; doi:10.1007/s00701-025-06669-3)
Supplement: Supplementary file 1 — Supplementary material 1 (PDF 482 KB) [file 701_2025_6669_MOESM1_ESM.pdf]

Title
Postoperative speech impairment and cranial nerve deficits in children undergoing posterior fossa tumor surgery with intraoperative MRI – a prospective multinational study

Journal Acta Neurochirurgica

Authors
Aske Foldbjerg Laustsen[1,2], Jonathan Kjær Grønbæk, Radek Frič, Shivaram Avula, Conor Mallucci, Pelle Nilsson, Per Nyman, Péter Hauser, Katalin Mudra, Rosita Kiudeliene, Saulius Ročka, Magnus Aasved Hjort, Rick Brandsma, Eelco Hoving, Andrea Carai, Vladimír Beneš, Jana Táborská, Christian Dorfer, Sandra Jacobs, Miriam Pavon-Mengual, Jane Skjøth-Rasmussen, Kjeld Schmiegelow, Astrid Sehested, René Mathiasen, Marianne Juhler

Corresponding author
Aske Foldbjerg Laustsen, e-mail: aske.foldbjerg.laustsen@regionh.dk
[1]Department of Neurosurgery, Rigshospitalet, Denmark
[2]Department of Paediatrics and Adolescent Medicine, Rigshospitalet, Denmark

Supplementary material

Supplementary Cross tables 1-4 postoperative cranial nerve deficit sum grouped and preoperative cranial nerve deficit sum

Supplementary Table 1 All patients

Preop. CND sum \ Postop. CND Group	0	1	2	3 or more	Not available
0	405	50	30	33	62
1	24	29	15	3	9
2	7	3	14	10	1
3	1	0	1	4	0
4	2	0	0	2	1
5	0	0	0	2	0
6	2	0	0	0	0
9	0	0	0	0	1
12	0	0	0	0	1
Not available	29	7	5	5	32

CND Cranial nerve deficit

Supplementary Table 2 Subgroup intraoperative MRI = no

Preop. CND sum \ Postop. CND Group	0	1	2	3 or more	Not available
0	259	42	19	20	37
1	11	21	12	3	7
2	5	3	8	6	0
3	1	0	1	4	0
4	2	0	0	0	1
12	1	0	0	0	0
Not available	0	0	0	0	1

CND Cranial nerve deficit

Supplementary Table 3 Subgroup Intraoperative MRI = yes

Postop. CND Group / Preop. CND sum	0	1	2	3 or more	Not available
0	79	6	8	5	14
1	6	5	0	0	1
2	2	0	0	1	1
Not available	4	1	1	1	6

CND Cranial nerve deficit

Supplementary Table 4 Subgroup Intraoperative MRI = Not available

Postop. CND Group / Preop. CND sum	0	1	2	3 or more	Not available
0	67	2	3	8	11
1	7	3	3	0	1
2	0	0	6	3	0
4	0	0	0	2	0
5	0	0	0	2	0
6	1	0	0	0	0
9	0	0	0	0	1
Not available	8	1	2	2	14

CND Cranial nerve deficit

Supplementary Table 5 Odds ratio results for POSI – sensitivity analysis with CND as a linear spline

**Univariate analysis**			**Multivariate analysis**			
**Postoperative speech impairment**			Model 1 (Intraoperative MRI and postoperative CND; n = 545)		Model 2 (Intraoperative MRI & postoperative CND & Tumour location; n = 525)	Model 3 (Intraoperative MRI & postoperative CND & Tumour location & age; n = 523)
Intraoperative MRI	OR (95% CI) (n = 602)	OR (95% CI) (Missing postop. CND removed) (n = 545)	OR (95% CI)	OR (95% CI) (Missing tumour location CND removed) (n = 525)	OR (95% CI)	OR (95% CI)
Yes	0.83 (0.53;1.30)	0.76 (0.46;1.27)	0.89 (0.53;1.50)	0.84 (0.49;1.46)	0.77 (0.44;1.34)	0.81 (0.45;1.43)
No	1 (ref)		1 (ref)		1 (ref)	1 (ref)
Postoperative CND (linear)	(n = 661)	(Missing ioMRI MR removed) (n = 545)				
0	1 (ref)		1 (ref)		1 (ref)	1 (ref)
1	2.91 (1.76;4.82)	2.84 (1.69;4.78)	2.81 (1.66;4.74)	2.85 (1.68;4.83)	1.91 (1.11;3.31)	1.87 (1.08;3.26)
2	2.37 (1.32;4.28)	2.41 (1.27;4.61)	2.40 (1.25;4.58)	2.75 (1.42;5.33)	2.04 (1.03;4.06)	2.09 (1.04;4.19)
3	2.05 (0.94;4.47)	1.92 (0.78;4.72)	1.92 (0.78;4.72)	2.35 (0.94;5.85)	1.81 (0.70;4.66)	1.85 (0.71;4.81)
>3	1.52 (1.21;1.92)	1.50 (1.16;1.94)	1.50 (1.15;1.94)	1.42 (1.09;1.84)	1.33 (1.02;1.73)	1.42 (1.09;1.86)

CI Confidence interval, *CND* Cranial nerve deficit, *MRI* Magnetic resonance imaging, *N* Number, *OR* Odds ratio, *POSI* Postoperative speech impairment

Supplementary Table 6 Odds ratio for risk of difference[a] in CND pre- and postoperative including adjustment for missing values

**Univariate analysis**		**Multivariate analysis**		
**Difference in post- and preoperative cranial nerve deficit**		Model 1 (preoperative CND; n = 530)		Model 2 (preoperative CND & Tumour location; n = 513)
Intraoperative MRI	OR (95% CI) (n = 530)	OR (95% CI)	OR (95% CI) (Missing tumour location removed) (n = 513)	OR (95% CI)
Yes	0.72 (0.45;1.14)	0.62 (0.38;0.99)	0.57 (0.35;0.92)	0.51 (0.31;0.83) p = 0.007
No	1 (ref)	1 (ref)		1 (ref)

[a]Difference group: Difference in sum of pre- and postoperative cranial nerve deficit. Cut off set to at -2 and 4, *CI* Confidence interval, *CND* Cranial nerve deficit, *MRI* Magnetic resonance imaging, *N* Number, *OR* Odds ratio, *Ref* Reference

Supplementary Table 7 Odds ratio results for postoperative CND and ioMRI including adjustment for missing values

**Univariate analysis**			**Multivariate analysis**			
**Postoperative cranial nerve deficit**			Model 1 (Preoperative CND, n = 530)		Model 2 (Preoperative CND & Intraoperative MRI & Tumour location; n = 328)	Model 3 (Preoperative CND & Intraoperative MRI & Tumour location & Country; n = 328)
**Intraoperative MRI**	OR (95% CI) (n = 563)	OR (95% CI) (Missing preoperative CND removed) (n = 530)	OR (95% CI)	OR (95% CI) (Missing tumour location removed) (n = 513)	OR (95% CI)	OR (95% CI)
Yes	0.63 (0.40;1.00)	0.59 (0.36;0.96)	0.67 (0.41;1.09)	0.61 (0.37;1.02)	0.58 (0.33;0.94) p=0.03	0.54 (0.29;0.98)
No	1 (ref)		1 (ref)	1 (ref)		1(ref)

CI Confidence interval, *CND* Cranial nerve deficit, *ioMRI* Intraoperative magnetic resonance imaging, *N* Number, *OR* Odds ratio, *Ref* Reference

Supplementary Table 8 Odds ratio results for risk of postoperative CND for countries utilizing ioMRI

**Univariate analysis**		**Multivariate analysis**	
**Postoperative cranial nerve deficit**		Model 1 (Preoperative CND, n = 394)	Model 2 (Preoperative CND & Tumour location; n = 383)
Intraoperative MRI	OR (95% CI) (n = 422)	OR (95% CI)	OR (95% CI)
Yes	0.67 (0.41;1.08)	0.67 (0.40;1.11)	0.52 (0.30;0.91) p=0.02
No	1 (ref)	1 (ref)	1 (ref)

CI Confidence interval, CND Cranial nerve deficit, ioMRI Intraoperative magnetic resonance imaging, *N* Number, OR Odds ratio, Ref Reference

Supplementary Table 9 Odds ratio results for POSI

Univariate analysis			Multivariate analysis					
**Postoperative speech impairment**			Model 1 (Intraoperative MRI and postoperative CND; n = 545)		Model 2 (Intraoperative MRI & postoperative CND & Tumour location; n = 525)	Model 3 (Intraoperative MRI & postoperative CND & Tumour location & age; n = 523)	Model 4 (Intraoperative MRI & postoperative CND & Tumour location & age & country; n = 523)	Model 5 (Intraoperative MRI & postoperative CND & tumour location & tumour type[a]; n = 481)
Intraoperative MRI	OR (95% CI) (n = 602)	OR (95% CI) (Missing postop. CND removed) (n = 545)	OR (95% CI)	OR (95% CI) (Missing tumour location CND removed) (n = 525)	OR (95% CI)	OR (95% CI)	OR (95% CI)	OR (95% CI)
Yes	0.83 (0.53;1.30)	0.76 (0.46;1.27)	0.85 (0.50;1.45)	0.80 (0.46;1.38)	0.73 (0.42;1.29)	0.76 (0.43;1.35)	0.76 (0.43;1.36)	0.80 (0.43;1.46)
No	1 (ref)		1 (ref)		1 (ref)	1 (ref)	1 (ref)	1 (ref)
Postoperative CND	OR (95% CI) (n = 661)	OR (95% CI) (Missing ioMRI MR removed)						
0	1 (ref)		1 (ref)		1 (ref)	1 (ref)	1 (ref)	1 (ref)
1	2.90 (1.75;4.78)	2.82 (1.68;4.76)	2.78 (1.65;4.70)	2.83 (1.67;4.78)	1.89 (1.09;3.27)	1.84 (1.06;3.20)	1.84 (1.06;3.20)	2.06 (1.15;3.68)
2	2.36 (1.31;4.26)	2.41 (1.26;4.59)	2.38 (1.23;4.55)	2.73 (1.41;5.28)	2.41 (1.09;5.33)	2.06 (1.03;4.13)	2.06 (1.03;4.13)	2.13 (1.02;4.42)
3 or more	5.39 (3.09;9.42)	4.77 (2.50;9.07)	4.72 (2.48;9.00)	5.16 (2.65;10.05)	3.53 (1.77;7.03)	4.13 (2.04;8.35)	4.13 (2.04;8.35)	4.15 (1.98;8.70)

[a]Pilocytic astrocytoma (reference), medulloblastoma, ependymoma, atypical teratoid/rhabdoid tumour, and other

CI Confidence interval, *CND* Cranial nerve deficit, *ioMRI* Intraoperative magnetic resonance imaging, *N* Number, *OR* Odds ratio, *Ref* Reference, *POSI* Postoperative speech impairment

Supplementary Table 10 Odds ratio results for POSI (countries utilizing ioMRI)

**Univariate analysis**		**Multivariate analysis**		
**Postoperative speech impairment**		Model 1 (postoperative CND; n = 410)	Model 2 (Postoperative CND & Tumour location; n = 397)	Model 3 (Postoperative CND & Tumour location & age; n = 395)
Intraoperative MRI	OR (95% CI) (n = 458)	OR (95% CI)	OR (95% CI)	OR (95% CI)
Yes	0.90 (0.56;1.44)	0.93 (0.53;1.62)	0.75 (0.41;1.36)	0.77 (0.42;1.41)
No	1 (ref)	1 (ref)	1 (ref)	1 (ref)

CI Confidence interval, *ioMRI* Intraoperative magnetic resonance imaging, *N* Number, *OR* Odds ratio, *Ref* Reference, *POSI* Postoperative speech impairment

Supplementary Figure 1 Barchart representing fraction of preoperative damage to individual cranial nerves; *L* Left, *R* Right

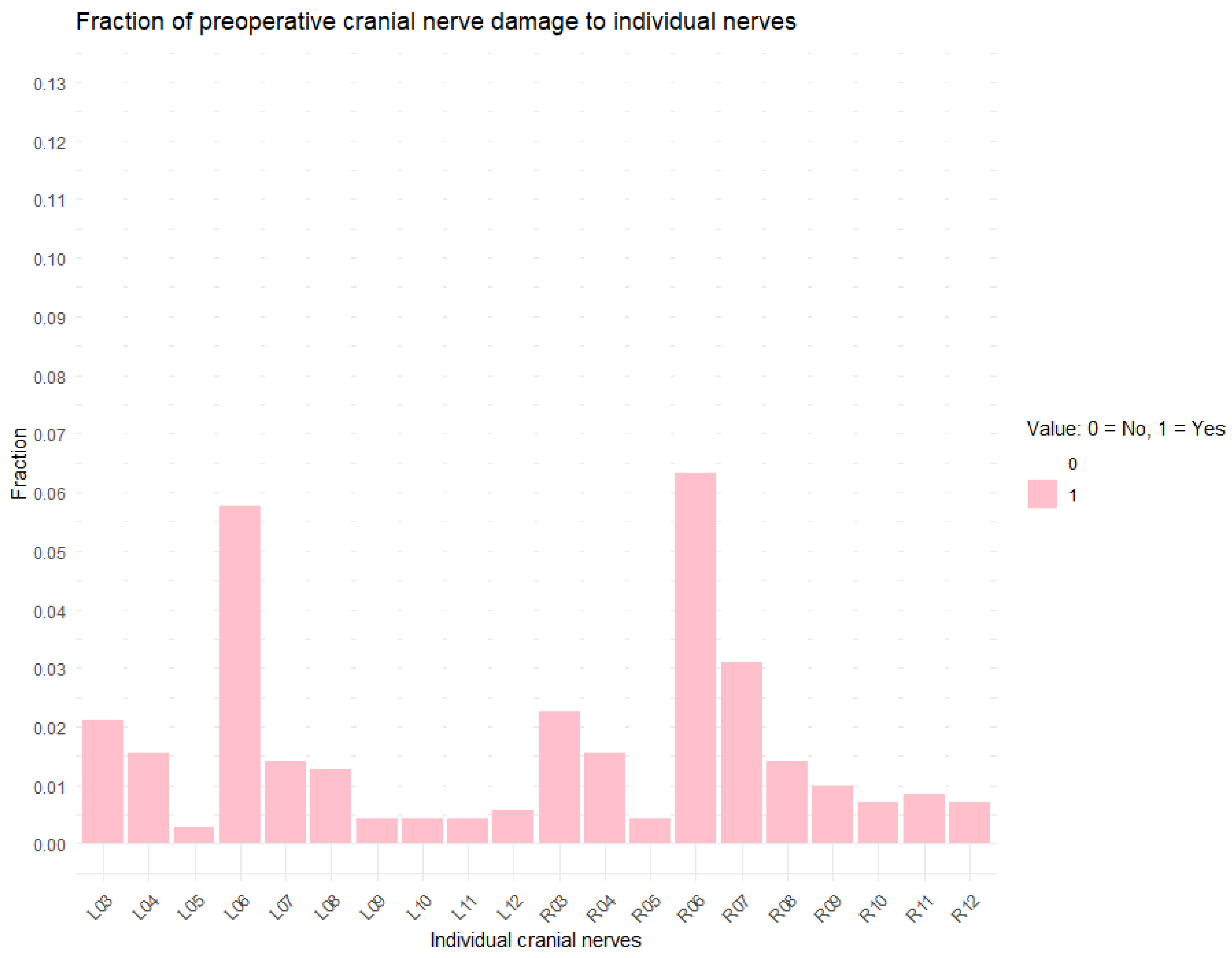

Supplementary Figure 2 Barchart representing fraction of postoperative damage to individual cranial nerves; *L* Left, *R* Right

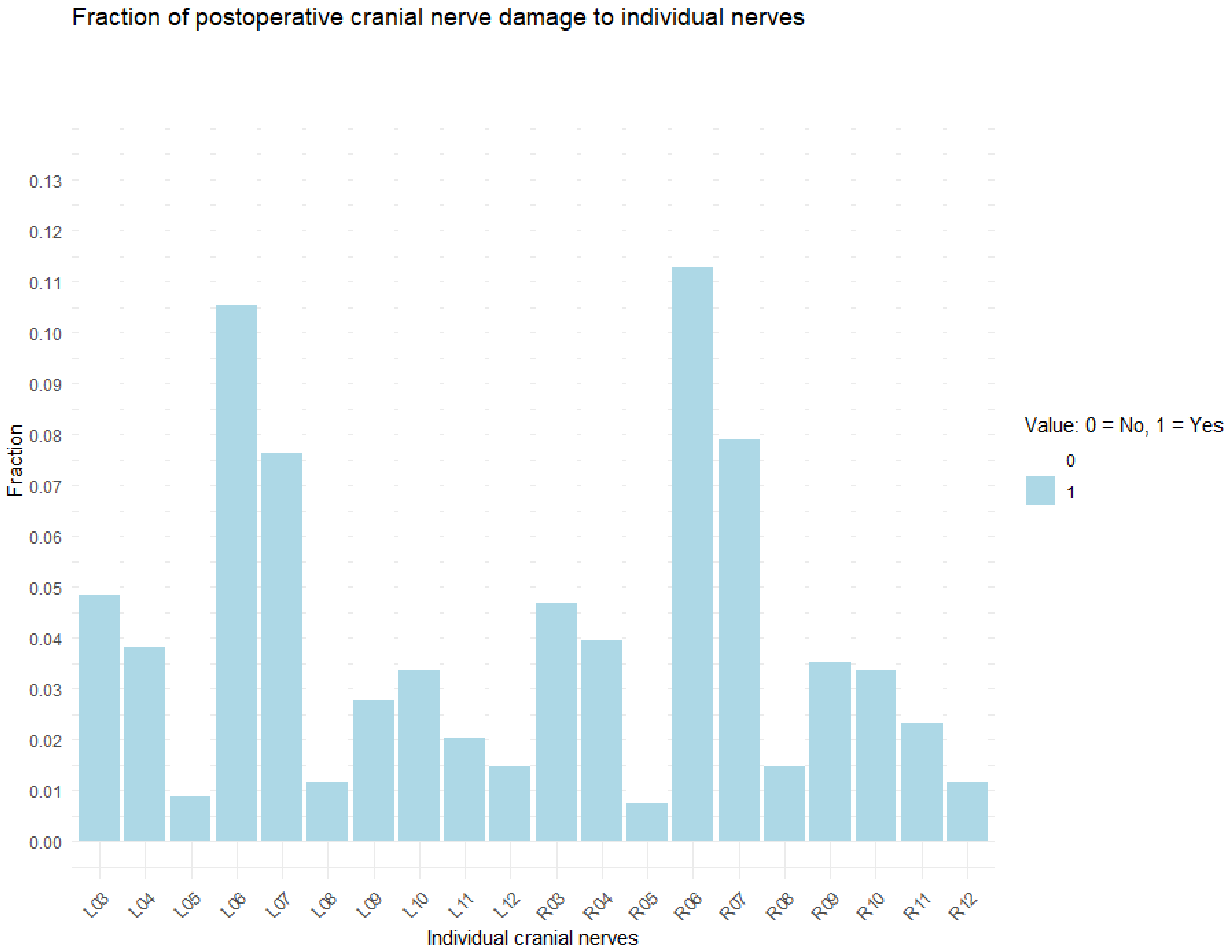

Supplementary Figure 3 Barchart of fraction of postoperative individual cranial nerve damage where presence of preoperative individual cranial nerve damage is set to "Not assessable"; *L* Left, *R* Right

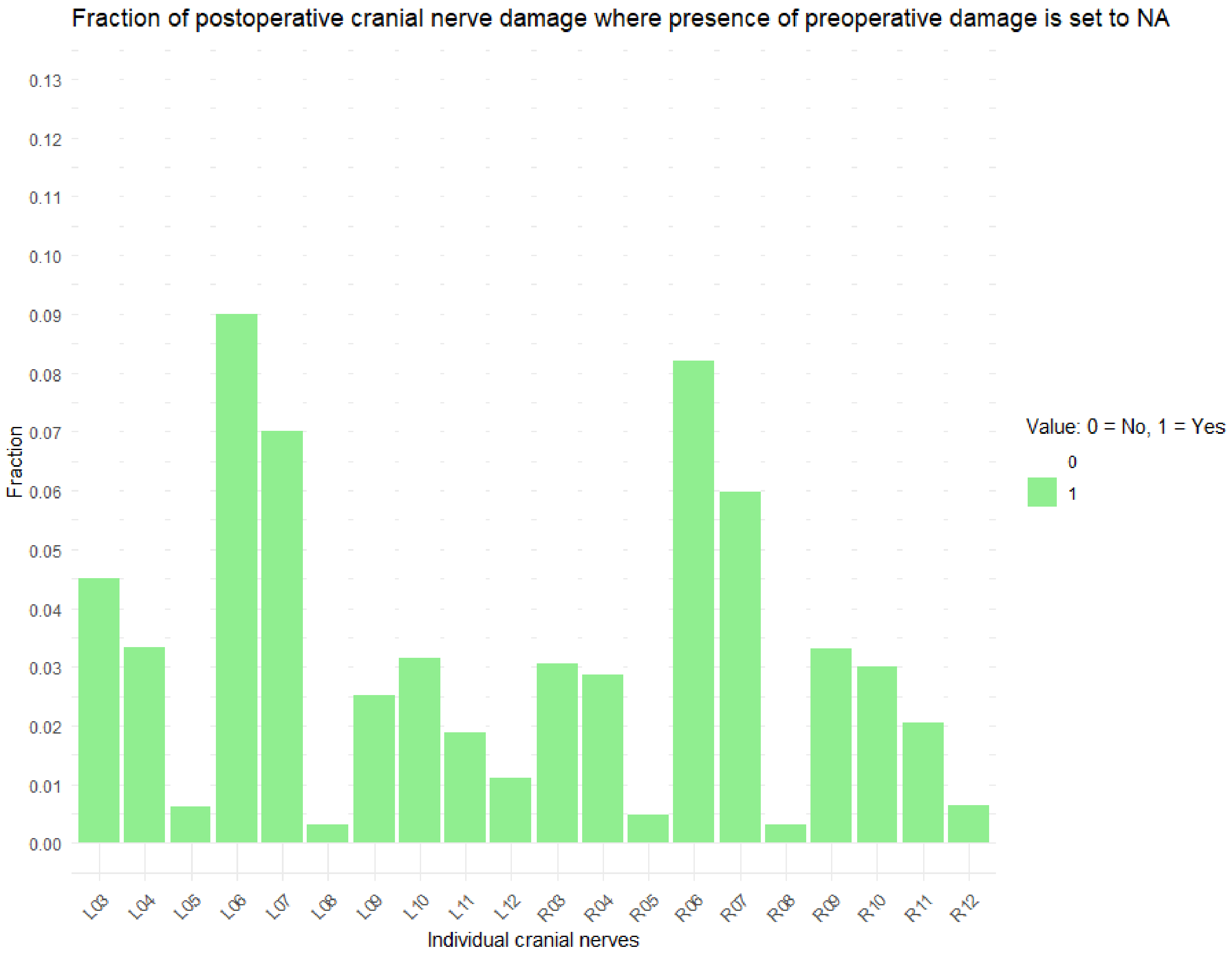

Supplementary Figure 4 Barchart of fraction of postoperative cranial nerve damage where absence of preoperative individual cranial nerve damage is set “Not assessable”; *L* Left, *R* Right

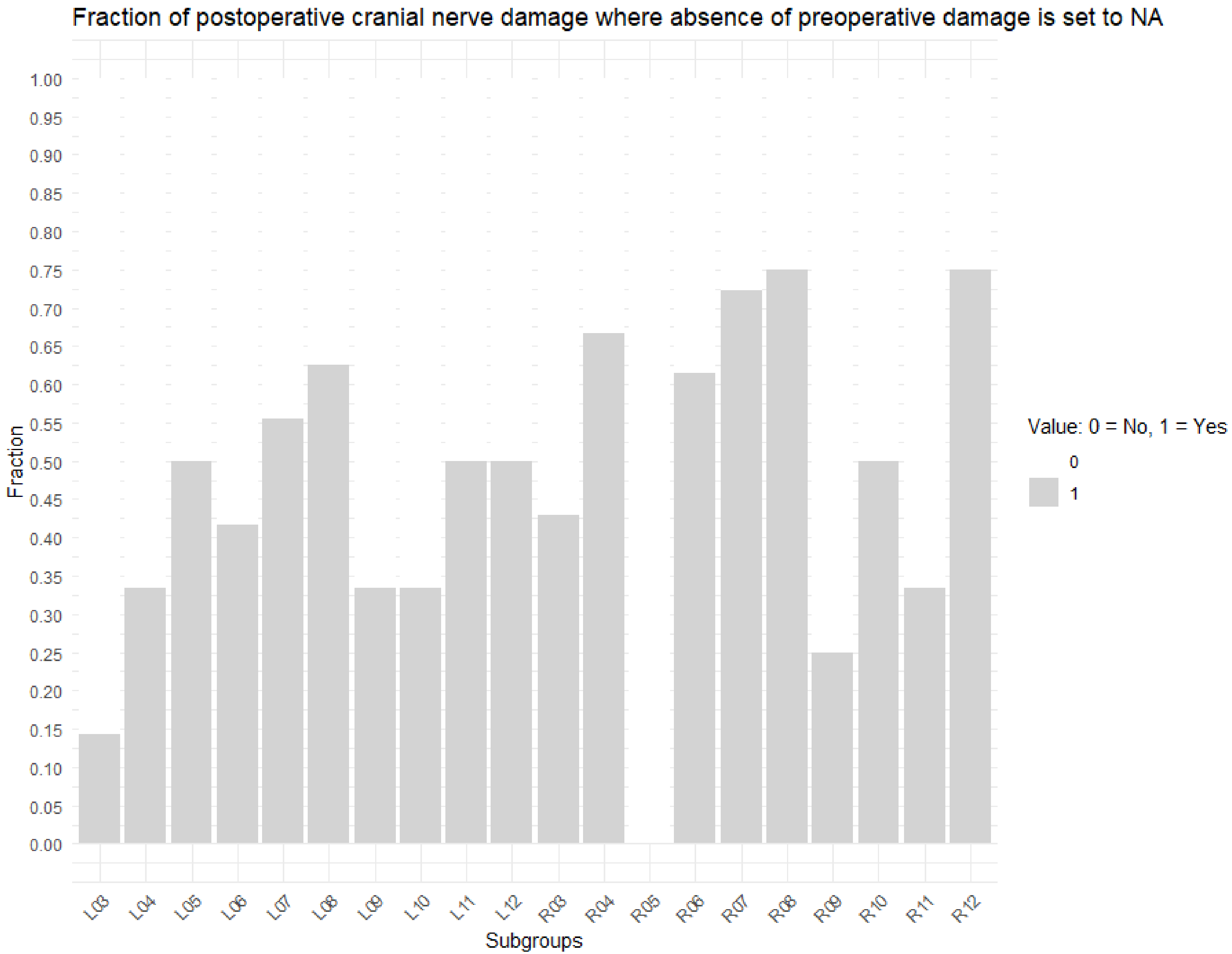

**Supplementary Table 11** Demographics	**All patients** (n = 790)			**Known status of Intraoperative MRI** (n = 643)		**Intraoperative MRI applied** (n = 141)	
	N	%[c]	%[d]	N	%[e]	N	%[f]
**Preoperative accumulated CND[a]**							
0	580	73.4	81.5	489	84	112	23
1	80	10.1	11.2	66	83	12	18
2	35	4.4	4.9	26	74	4	15
3	6	0.8	0.8	6	100	0	0
4	5	0.6	0.7	3	60	0	0
5	2	0.3	0.3	1	50	0	0
6	2	0.3	0.3	0	0	0	-
9	1	0.1	0.1	0	0	0	-
12	1	0.1	0.1	1	100	0	0
Unknown	78	9.9		51	65	13	26
**Postoperative accumulated CND[b]**							
0	470	59.5	68.8	387	82	91	24
1	89	11.3	13.0	83	93	12	14
2	65	8.2	9.5	51	78	9	18
3	22	2.8	3.2	16	73	4	25
4	9	1.1	1.3	8	89	1	13
5	6	0.8	0.9	4	67	0	0
6	8	1.0	1.2	5	63	1	20
7	3	0.4	0.4	1	33	0	0
8	4	0.5	0.6	4	100	1	25
10	4	0.5	0.6	3	75	0	0
12	1	0.1	0.1	1	100	0	0
13	1	0.1	0.1	0	0	0	-
18	1	0.1	0.1	0	0	0	-
Unknown	107	13.5		80	75	22	28

[a]Accumulated damaged CN's preoperatively, [b]Accumulated damaged CN's postoperatively, [c]all patients eligible for analysis (*vertical*), [d]% among patients with known CND status, [e]% of patient characteristics or tumour characteristics (*horisontal*), [f]% of subgroup with known status of intraoperative MRI (*horisontal*)
CND Cranial nerve deficit, *MRI* Magnetic resonance imaging, *N* Number

**Supplementary Table 12** Demographics	**Habitual speech** (n = 551)		**Reduced speech** (n = 98)		**Mutism** (n = 85)	
	**N**	**%[a]**	**N**	**%[b]**	**N**	**%[b]**
**Postoperative CND**						
0	383	52	50	11	24	5
1	54	7	17	20	13	15
2	45	6	8	13	11	17
3 or more	28	4	13	23	15	27
Unknown	41	5[c]	10	14	22	30

[a]% of known speech status, [b]% in CND category, [c]% of total cohort (n = 790)
CND Cranial nerve deficit, *N* Number
